# Sialolipoma of the parotid gland: A rare entity. Case report

**DOI:** 10.1016/j.ijscr.2024.109264

**Published:** 2024-01-13

**Authors:** Adrián Torres-Parlange, Emma Karina Martínez-Cárdenas, Oscar Blancarte-Vidal, Yosef Dueñez-Uriarte, Alina Melissa González-Ortiz, Quitzia Libertad Torres-Salazar

**Affiliations:** a Hospital General 5 de diciembre, Mexicali, Baja California, Mexican Institute of Social Security, Mexico; b Universidad Juárez del Estado de Durango, Facultad de Medicina y Nutrición, Mexico

**Keywords:** Sialolipoma, Parotid gland, Case report, Salivary gland tumor

## Abstract

**Introduction and importance:**

Lipomatous neoplasms of the parotid gland represent an exceptionally rare and often underdiagnosed category of tumors, accounting for an incidence ranging from 0.6 % to 4.4 % of all neoplasms detected within the parotid gland. Sialolipoma is defined as an uncommon variant of lipoma, characterized by a well-defined proliferation of mature adipocytes with secondary entrapment of salivary gland elements, including serous acini, ducts, and myoepithelial cells.

**Case presentation:**

The current case pertains to a 17-year-old female who presented with a one-year history of enlargement in the left preauricular region.

**Clinical discussion:**

The case we present poses a complex diagnostic challenge due to two distinct characteristics. The diagnostic challenge lies in its remarkably low incidence and the propensity for confusion with pleomorphic adenoma, which is the most common tumor of the parotid gland. It is a benign disease entity characterized by the absence of dysplasia, in marked contrast to pleomorphic adenoma.

**Conclusions:**

The infrequency in the manifestation of these tumor types, coupled with their prolonged asymptomatic course, can pose a diagnostic challenge. Enhancing our knowledge to comprehensively delineate these entities is imperative to effectively address the diagnostic complexities from both clinical and histopathological standpoints.

## Highlights

• Cases of sialolipoma in the parotid gland stand out for their diagnostic complexity.
• The presence of mature adipose tissue and non-neoplastic salivary gland elements is emphasised.
• It may serve as a guide for future diagnoses.

## 1 Introduction

Salivary gland tumors make up a small proportion, less than 3 %, of all neoplastic lesions affecting the craniocervical region. The parotid gland is the primary site of incidence for these tumors, whether they are benign or malignant. Although most of these tumors originate from epithelial tissue, about 2–5 % arise from mesenchymal cells [1]. Based on the reviewed literature, very few cases have been documented globally. Most of these cases (51.4 %) have specifically originated in the parotid gland [2]. The reported symptoms in these cases mimic those of a pleomorphic adenoma, with a significantly lower incidence of complications related to facial venous entrapment (14 %) [[3], [4], [5]].

Sialolipoma represents a particular histological variant of lipoma, first documented by Nagao et al. in 2001 [6]. It appears in the 2005 World Health Organization (WHO) classification and has been incorporated as a separate entity in the most recent 2017 WHO classification of head and neck tumors. This tumor type typically manifests as a well-defined mass with a fibrous envelope, composed of neoplastic mature adipose tissue and non-neoplastic salivary gland components [7].

From a clinical perspective, the sialolipoma typically manifests as a precisely delineated localized swelling. Its observed maximum diameter can extend up to 3 cm, displaying a soft consistency and a yellowish appearance. In certain instances, when associated with traumatic factors, its surface may adopt a yellow-white tint. Submersion in formalin results in the characteristic floating of the surgical specimen, indicating the presence of adipose content within its structure. Initially, it is often perceived as a glandular anomaly akin to a pleomorphic adenoma. Accurate diagnosis is only achievable through histopathological examination. When involving the parotid gland, it may incite facial paralysis. Surgical excision stands as the primary therapeutic approach, with no reported instances of recurrence documented to date [8].

Lipoadenoma shares significant histological similarities with sialolipoma. The main distinction is that lipoadenoma consists of adipose tissue and ductal components, whereas sialolipoma, in addition to these elements, contains glandular acini. We present a case report of a 17-year-old female with a diagnosis of parotid sialolipoma. The work has been reported in line with the SCARE criteria [9].

## 2 Clinical case presentation

The case involves a 17-year-old female patient who presented for consultation due to the identification of a mass in the left preauricular region persisting for 1 year. On palpation, a soft, mobile, and painless tumor measuring 2 cm in diameter was noted. This enlargement was associated with paresthesias in the left cheek, the sole reported symptom. The patient had a history of percutaneous drainage of a similar lesion in the same region at 8 years of age, which showed a favorable outcome. No other pertinent medical history was identified. Considering the clinical presentation, comprehensive laboratory tests revealed no pathological findings, prompting an excisional biopsy of the tumor. A 3 cm longitudinal incision was made over the tumor, which was subsequently dissected and excised completely. The resulting wound was closed using nylon material in a simple interrupted suture technique. A sample was sent for pathological analysis.

**Image 1:**
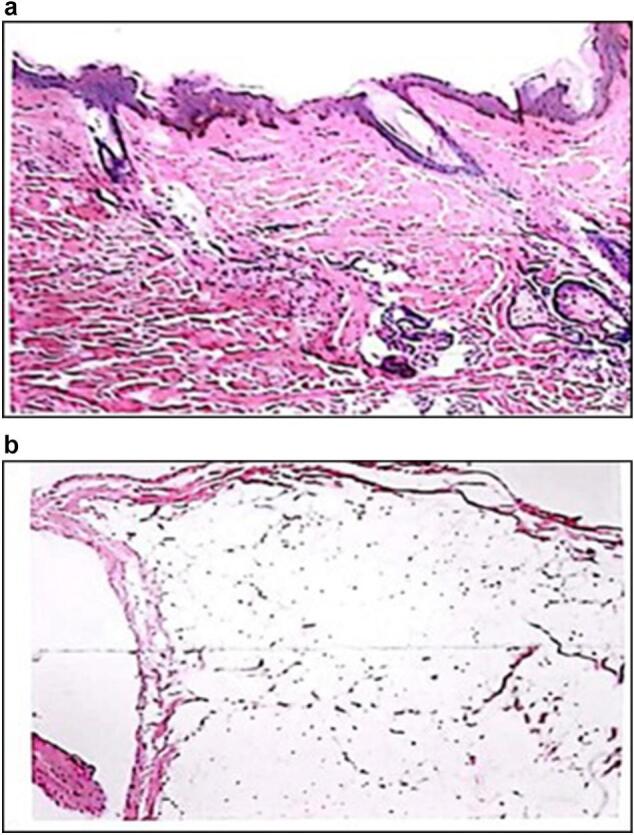
a and b: Excisional biopsy of nodule in left parotid region.

**Image 2:**
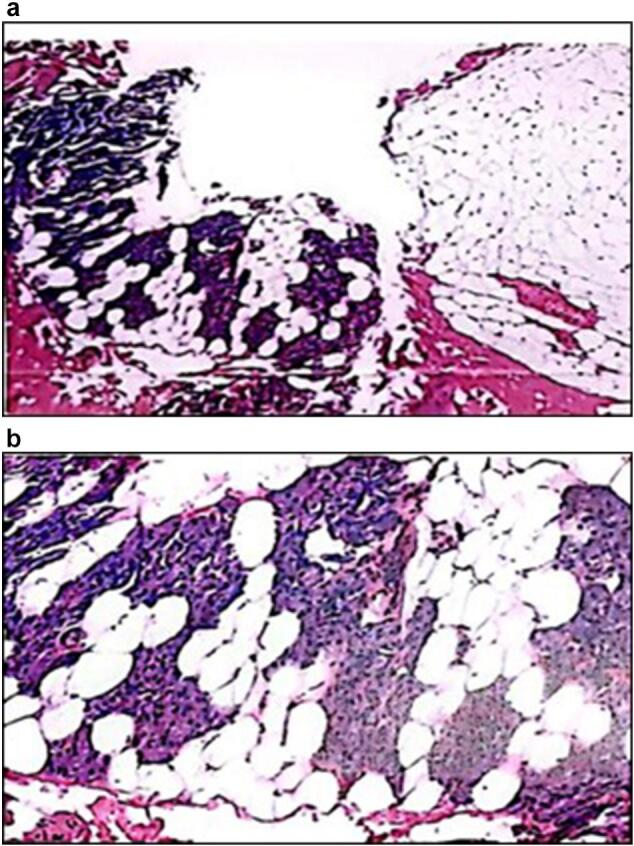
a and b: Fragments of benign mesenchymal neoplasm of adipose lineage with lobulated contour. The proliferation of adipocytes stands out.

The macroscopic examination of the specimen exhibited dimensions of 4 × 3 × 0.3 cm and displayed preserved, soft, greyish-yellow, granular tissue for detailed microscopic analysis (Image 1a and b). Microscopic evaluation revealed an unaltered epidermis in underlying connection with fragments of a benign mesenchymal neoplasm of adipose origin with lobulated contours. This neoplasm demonstrated a proliferation of adipocytes with minimal variations in shape and size, resembling mature adipocytes, and lacked septa. Furthermore, transitions with serous glandular acini of the parotid gland were identified, and the lobules were demarcated by adipose tissue with similar characteristics (Image 2a and b). Ultimately, the diagnosis of sialolipoma in the left parotid gland was confirmed. The patient was discharged on the same day. A one-week follow-up revealed substantial improvement without any complications. Concerning the initial clinical manifestation of paresthesias, the patient persisted in experiencing paresthesias in the left cheek for the two weeks subsequent to the surgical procedure, coupled with a mild loss of movement in the left labial commissure. At the two-week mark post-surgery, the patient became asymptomatic. Throughout a one-year follow-up period, no indications of recurrence were noted.

## 3 Discussion

The case we present poses a complex diagnostic challenge due to two distinct characteristics. Firstly, the higher incidence of sialolipomas has been reported in the male population [3] Secondly, the occurrence of paresthesia in the left cheek as the sole symptom, without any accompanying medical history or pathological indications, added further complexity to the diagnosis. Furthermore, the age factor contributed to the decreased suspicion, as reported by Kidambi et al., where 90 % of documented sialolipoma cases were observed in adult individuals over 18 years of age. Notably, three cases of sialolipoma were documented in patients under the age of 5. Additionally, only two cases of congenital sialolipoma were identified [10]. These instances comprised one case in a patient at birth and the other in a 6-week-old male infant, with both cases showing sialolipoma in the parotid gland [10,11].

The etiology of sialolipoma remains not completely elucidated. Some researchers suggest that the pathogenesis of sialolipoma might be associated with salivary gland malfunction, leading to an altered configuration of the salivary glands. This microscopic alteration is characterized by the substitution of normal salivary gland tissue with mature adipose tissue, accompanied by atrophic salivary glandular elements, chronic changes in ductal epithelial cells such as oncocytic metaplasia, fibrosis, and lymphocytic infiltration [12]. Microscopically, sialolipomas are typically recognized as well-demarcated, often encapsulated masses comprising benign neoplastic adipose tissue interspersed with foci of acini and usually atrophic, non-neoplastic salivary gland ducts within the lipomatous proliferation. Adipose tissue accounts for 90 % of sialolipomas in the parotid gland and 50 % of cases affecting the palate [6]. In the presented case, the microscopic examination details the presence of fragments of benign neoplasia characterized by adipocyte proliferation.

Finally, the history of percutaneous drainage of a similar lesion in the same region, with a favorable evolution, is another noteworthy aspect, as cases of recurrence in this type of tumor have been described in extremely rare cases [13].

## 4 Conclusions

The patient presented with a sialolipoma in the left parotid gland, representing a rare subtype of tumor distinguished by its unique clinical presentation and histopathological features. The exclusive occurrence of paresthesias in the left cheek adds intricacy to the clinical scenario. Notably, the history of prior percutaneous drainage of a similar lesion in the same region, with a favorable outcome, is significant, particularly since recurrence in this particular tumor type has not been reported. Finally, the comprehensive macroscopic and microscopic analysis of the specimen offers a comprehensive comprehension of the nature and attributes of the sialolipoma, significantly contributing to the knowledge and comprehension of this uncommon condition.

## Parental consent for minors

Written informed consent was obtained from the patient's parents/legal guardian for publication and any accompanying images. A copy of the written consent is available for review by the Editor-in-Chief of this journal on request.

## Ethical approval

This study does not require committee approval because it is a case report; however, we have the patient's authorization to use this material for publication.

## Funding

Nothing to declare.

## Author contribution

ATP - Diagnosis and follow-up

EKMC - Surgical approach plan

OBV - Surgical assistant

YDU - File tracking and documentation

AMGO - Bibliographic review

QLTS - Article redaction

## Guarantor

Quitzia Libertad Torres Salazar.

## Conflict of interest statement

Nothing to declare.
